# Project SATURN– a real-world evidence data collaboration with existing European datasets in Osteogenesis Imperfecta to support future therapies

**DOI:** 10.1186/s13023-024-03185-y

**Published:** 2024-05-02

**Authors:** L. Sangiorgi, M. Boarini, I. Westerheim, R. T. Skarberg, J. Clancy, V. Wang, M. Mordenti

**Affiliations:** 1 Department of Rare Skeletal Disorders, IRCCS Istituto Ortopedico Rizzoli, Bologna, Italy; 2 OIFE (Osteogenesis Imperfecta Federation Europe), Heffen (Mechelen), Belgium; 3 ePAG ERN BOND (European Reference Network on Rare Bone Diseases), Coordinating Center, Bologna, Italy; 4 Mereo BioPharma Group plc, London, UK; 5 UBC Late Stage Ltd, London, UK

**Keywords:** rare diseases, osteogenesis imperfecta, real-world data, health technology assessment, regulators, healthcare systems, registries, common core dataset, natural history

## Abstract

Regulatory marketing authorisation is not enough to ensure patient access to new medicinal products. Health Technology Assessment bodies may require data on effectiveness, relative effectiveness, and cost-effectiveness. Healthcare systems may require data on clinical utility, savings, and budget impact. Furthermore, the exact requirements of these bodies vary country by country and sometimes even region to region, resulting in a patchwork of different data requirements to achieve effective, reimbursed patient access to new therapies. In addition, clinicians require data to make informed clinical management decisions. This requirement is of key importance in rare diseases where there is often limited data and clinical experience at the time of regulatory approval.

This paper describes an innovative initiative that is called Project SATURN: **S**ystematic **A**ccumulation of **T**reatment practices and **U**tilization, **R**eal world evidence, and **N**atural history data for the rare disease Osteogenesis Imperfecta. The objective of this project is to generate a common core dataset by utilising existing data sources to meet the needs of the various stakeholders and avoiding fragmentation through multiple approaches (e.g., a series of individual national requests/approaches, and unconnected with the regulators’ potential requirements). It is expected that such an approach will reduce the time for patient access to life-changing medications. Whilst Project SATURN applies to Osteogenesis Imperfecta, it is anticipated that the principles could also be applied to other rare diseases and reduce the time for patient access to new medications.

**Supplementary Information:**

The online version contains supplementary material available at 10.1186/s13023-024-03185-y.

## Background

Despite initiatives over the last 20 years to encourage the development of medicines for rare diseases (RDs), thousands of diseases remain where the medical need is unmet. Estimates of the number of affected individuals vary in detail but suggest as many as 446 million worldwide and 30 million in Europe [1]. An estimated 10% of RDs currently have a medical treatment option, leaving 90% without [2].

The European Union’s (EU) Regulation 141/2000 [3] on orphan medicinal products states that, “patients suffering from rare conditions should be entitled to the same quality of treatment as other patients” and “sponsors of orphan medicinal products designated under EU Regulation 141/2000 should be entitled to the full benefit of any incentives granted by the Community or by the Member States to support the research and development of medicinal products for the diagnosis, prevention or treatment of RDs”.

There is evidence that this regulation has had a measure of success because in the two decades following its implementation, 150 new medicinal products were approved for RDs compared to only eight prior to the regulation coming into effect. However, success in this context is defined as “regulatory approval” and this does not equate to patient access to new therapies. A total of 143 orphan medicinal products were reviewed that had obtained marketing authorisation in the EU between 2000 and the end of 2016, and found that while over 50% of these were centrally authorized in the five largest EU economies of the then-28 EU Member States, the various national reimbursement policies have created further limitations to patient access [4].

Patients with RDs face enormous challenges in diagnosis, accessing specialist care, ensuring quality of life, and accessing therapies that are more targeted to the condition [5]. Patient benefit from therapies can only be achieved if the regulators approve the treatment, healthcare systems are willing to pay, and the prescribers are willing to prescribe.

Patients with RDs share the common challenges around getting new therapies approved by European regulators and national authorities, as well as accessing therapy in their local healthcare system. Stakeholders within the area of RD share a common goal of providing timely and sustainable availability of therapies. For this to happen, all stakeholders need to work together to find solutions to overcome the barriers that come with any RD (e.g., lack of documentation, smaller patient populations, and smaller markets for medicinal products that are intended to treat patients with rare diseases). Additionally, it is the shared responsibility among RD stakeholders to secure sustainable pricing and thereby enable access to new treatments by the patient communities after authorization.

In the case of RDs, where not only patients are rare but also expertise is scarce and scattered, gathering sufficient data to inform effective decision-making can be challenging. Clinical trials are often of short duration, sometimes with novel designs; this leads to a lack of solid evidence on which to make valued assessments or pricing and reimbursement decisions, compared to treatments for more prevalent conditions. Data on natural history, disease evolution, and treatment outcomes, even in the absence of an on-label therapy, may be gathered in different individual centres in different countries, mandated either by national governments or as part of the research and knowledge-building efforts by specialised treating physicians in a given RD or therapeutic area.

In this paper, we describe the innovative initiative of Project SATURN: **S**ystematic **A**ccumulation of **T**reatment practices and **U**tilization, **R**eal world evidence and **N**atural history data in the rare disease of Osteogenesis Imperfecta (OI) and the development of a core dataset aiming to address the needs of all stakeholders utilising existing sources, with primary data remaining at source. Project SATURN is funded by Mereo BioPharma with collaboration between stakeholders from OI patient association (Osteogenesis Imperfecta Federation Europe, OIFE), European virtual network for RD (European Reference Network on Rare Bone Diseases, ERN BOND) and researcher (IRCCS Istituto Ortopedico Rizzoli - IOR).

## The osteogenesis imperfecta community

Unlike many RDs, OI has a well-established, well-structured, and well-connected community with a high level of awareness. Patient groups have been in existence for many years including the Brittle Bone Society formed in the United Kingdom (UK) in 1968 [6], the OI Foundation in the United States (US) in 1970 [7] and more recently, in 1993, the European umbrella for OI organizations in Europe– the OI Federation Europe (OIFE) [8].

For OI in some countries, there are well-established core centres of expertise and treatment that are led by physicians in partnership with patient representatives. These centres have existing and well-established data collection systems for patients, from formal registries to site-specific organised data collection systems (e.g., Registry of Osteogenesis Imperfecta in Italy, Norwegian Registry of Rare Congenital Bone Diseases, Brittle Bone Disorders Consortium in US, Centres of excellence in Cologne, Madrid, Sheffield and Utrecht). There are also federated registries, such as EuRR-Bone (European Registries for Rare Bone and Mineral Conditions) where all European centres working with OI and other rare bone and mineral conditions can provide data. EuRR-Bone was initially a three-year project partially funded by EU Health Programme and is now part of the European Reference Network on Rare Bone Diseases (ERN BOND) actions.

All of these considerations make OI a good candidate for the development of Project SATURN. However, it is intended that the general principles could be extended to other RDs.

## The need for a common core dataset

Inevitably in RDs, clinical trial data is based upon a small, selected patient population over a limited period of time. This data may be sufficient to achieve marketing authorisation but is unlikely to be sufficient to provide the necessary data required for evaluations to achieve reimbursed and equitable patient access across all countries where marketing authorisation has been obtained [9–11]. Regulatory approval is based upon data demonstrating Quality, Safety, and Efficacy. Health Technology Assessment (HTA) bodies may require data on Effectiveness (extent to which an intervention does more good than harm, under usual circumstances of health care practice), Relative Effectiveness (extent to which an intervention does more good than harm when under ideal circumstances, compared to alternative interventions), and Cost-Effectiveness. Healthcare systems may require data on Clinical Utility, Savings, and Budget Impact. Furthermore, the exact requirements of these bodies varies, leading to a patchwork of different data requirements to achieve effective, reimbursed patient access to new therapies.

Additionally, the regulatory requirement for data for medicinal products particularly in RDs does not stop at regulatory approval. In order to expand on the data available at the time of marketing authorisation, there is often the need for post-authorisation safety studies (PASS) or post-authorisation efficacy studies (PAES) requested as part of the granting of a Marketing Authorisation [12, 13]. The likelihood of these follow-up requests from the Regulatory agencies is higher in the case of uncertainty at the time of marketing authorisation where there is a high unmet medical need, but where sufficient data on both efficacy as well as safety might need to be developed. This is often the case in RDs.

Early in drug development, the need to understand the natural history of a disease is critical. This will inform the clinical development plan. Also, particularly in RDs where it may not be feasible or ethical to include a placebo-controlled arm in a clinical study, high quality natural history data may be accepted by both the United States Food and Drug Administration (FDA) and the European Medicines Agency (EMA) as an alternative [14, 15].

Clinical data is the cornerstone for diagnosis and management of OI patients. A core dataset would be valuable in supporting clinicians and researchers in the management of OI patients. It would also inform healthcare systems about the real-life clinical contribution of a new therapeutic intervention once a treatment has been made available.

The various stakeholders require different data; navigating these requirements, identifying data sources, and generating the data can potentially delay patient access to these much-needed therapies, which runs counter to the objective of the EU Orphan Regulation. By generating a common core dataset to pro-actively meet the needs of the various stakeholders, it is anticipated that decisions about access to therapies in RDs will be more rapid.

## The concept of project SATURN

The underlying principle of Project SATURN is a prospectively designed, integrated evidence generation plan based upon existing OI datasets in national centres of expertise, based on dialogue with the data customers, be they regulators, HTAs, and/or healthcare systems. It is not the intention of Project SATURN to create a new stand-alone registry. To do so would be sub-optimal to what already exists, would be duplicative in terms of cost, time and burden, and would not be welcomed by either physicians or patients. Additionally, it would be contrary to the principles set out in the EMA guidance on Registry-based studies [16], which encourages the use of existing data collection programmes.

The objective is to work with existing datasets, within a framework that corresponds to what the data-customers (e.g., regulators, HTAs, healthcare systems) will seek, avoiding fragmentation through multiple approaches (e.g., a series of individual national requests/approaches and unconnected with the regulators’ potential requirements) and supporting equity of access to potential new therapies for OI. This collaboration is not intended to formally link the existing registries nor to create an over-arching meta-registry. It is, rather, to develop a common core dataset for OI which would meet the requirements of the many stakeholders with data-ownership still remaining at source in the hands of those who originally collected it and will continue to collect it.

### Development of core dataset

The approach of Project SATURN is to establish a collaborative common core dataset, corresponding to the main data fields of relevance to OI which all independent datasets are broadly collecting (as per ‘the set of European common data elements for RD registrations’ [17]). The proposed common core dataset will be tested and refined during the process, including with future data customers, (e.g., regulators, HTAs, healthcare systems), who will have an opportunity to review and comment on the planned common core dataset. By prospectively designing and seeking input, the aim is for the resulting dataset to be able to meet their requirements. This will be done in collaboration with all stakeholders: physicians, registry/dataset owners, patients, OI community leaders, EU policymakers, regulators, HTAs, and healthcare systems. The intention is to prospectively design and work with the data customers (e.g., regulators, HTAs, healthcare systems) to understand what the likely future data gaps/evidence requirements will be and build the common core dataset based on these as far as possible.

The draft data set will be refined as Project SATURN progresses and there is an understanding of the variables that can be collected in existing datasets. Additionally, if datasets owners are in agreement, they can add further data collection variables to their datasets to meet the shared variables.

### Potential challenges

The key potential challenge with this approach is that all existing datasets consist of data that has been entered by healthcare providers in the course of their treatment of OI under conditions of routine clinical practice, rather than under the more stringent conditions of a clinical trial setting. Additionally, these data collection systems rely on healthcare providers voluntarily entering data. As a result, there is likely potential for missing data.

Across different countries, standards of care in the management of OI may vary. ERN BOND has a work package to map existing Clinical Practice Guidelines for Rare Bone and Mineral Disorders used by ERN BOND members, to determine the need for harmonisation and to update currently used guidelines, focusing on OI (Work Packages– ERN BOND) [18]. However, new clinical practice guidelines are not adopted overnight and there remains the potential for data elements to be non-standardised across the various datasets (i.e. quality of life questionnaires). Creating recommendations across ERN BOND members is an important steppingstone in this process.

Consultation with key stakeholders is crucial for the development of the core dataset. However, there remains the possibility that a stakeholder may require data to be collected that is not in the existing dataset. This is particularly the case for adverse event data collection, which may not always be captured, especially when the focus of the data collection is clinical outcomes. Post-Authorisation Safety Studies, by definition, will require the collection of adverse event data for future new therapies for OI.

The key to the success of this project is proactive consultations with stakeholders on an ongoing basis to define the requirements of each, in order for the core data set to be defined, integrated into clinical practice in the future and, thus, allow key data to be uniformly collected across the various datasets in a timely manner to hasten access of medical products to patients with RDs. Appropriate forums or opportunities to do so may not always exist and may need to be sought out or proactively identified by all parties.

### Pilot study

The Registry of Osteogenesis Imperfecta (ROI) database at IOR in Italy is a well-established dataset [19], gathering records between 600 and 700 patients with OI over more than 10 years using a robust and validated cloud-based electronic platform. This database was evaluated as the pilot by conducting a variable gap analysis (to assess what data variables are collected by the registry) and by collecting responses on the Registry Evaluation of Quality Standards Tool (REQueST), developed by the European Network for Health Technology Assessment (EUnetHTA). The standards set out in the tool are universal and essential elements of good practice and evidence quality that are relevant for different types of registries [20].

The variable gap analysis was completed by reviewing each variable in the ROI Case Report Form (CRF) against a list of critical variables (assessments, fracture collection and history, treatment for OI, treatment for pain, registry consent, safety, quality of life, patient characteristics, health resource utilization) based upon clinical trial standards and anticipated post-authorisation safety study requirements which is anticipated to be important for data customers (e.g., regulators, HTAs, healthcare systems).

REQueST was completed by IOR providing a comprehensive overview covering important aspects relating to the data quality and quality of the registry.

Pseudonymized patient data, in accordance with national and European regulations, will be extracted by IOR and provided in an aggregated format of Tables, Listings, and Figures (TLFs), ensuring the European General Data Protection Regulation is followed (https://gdpr-info.eu/). The specific TLFs to be sent in aggregate format will be based on the variables available as noted in the variable gap analysis. The results will be shared with stakeholders through Mereo BioPharma.

As project SATURN progresses, there will be further data collections or transfers allowing the possibility to add additional aggregated data from future follow-up visits to the dataset.

The initial transfer will be a natural history cut. It is hoped that as further data is collected and the shared data variables defined and complete, that longitudinal data will be collected from the collaborations to answer research questions in the future.

The initial pilot for project SATURN is planned for completion in 2024.

### Further collaboration to include datasets in other European countries

The same process (variable gap analysis and REQueST assessment) will then be repeated with other data-gathering centres across Europe to ensure they are sufficiently robust. This will secure a broad coverage including different countries as well as to maximise the value of interrogating different OI datasets to aggregate data (build bigger numbers). There is funding available for the collaborators’ time and data collection. The approach to assisting each registry or site will be adapted based on the existing dataset. In some cases, the same approach as with the IOR can be taken. In other cases, where a smaller, less formal dataset is available, a retrospective chart review may be a more appropriate methodology. However, regardless of the data abstraction methodology (chart review, aggregate TLF transfer), all data will be assessed in the same way (using the core dataset for variable gap analysis and REQueST assessment) by Mereo BioPharma and all data collected will remain at source within the community.

This approach will be applied gradually in more countries to build a European dataset by seeking willing partners and collaborators centre by centre, and at the same time, consistently and rigorously applying a methodology to ensure consistency and quality of data outputs. This is ensured by the consistent feasibility, dataset analysis, and data mapping review stages against the core common dataset, which will be reviewed and refined at each stage.

Project SATURN also plans to support and enhance local data collection. If the local dataset team sees value in expanding their data collection to align with the collaborative core dataset, the further enhancement of local data collection will be supported. Not all local data collection centres will necessarily have data on all OI aspects, given the broad range of fields that could be tracked. It is possible that, if a regulatory authority (HTA or a payer) requests information on a specific aspect of OI, that a limited number of centres within the future network of collaborating centres could provide data to answer that given question.

Further collaborations are being assessed to begin work in 2024.

## Conclusion

Project SATURN intends to respond to the data needs of multiple stakeholders to secure timely, sustainable, and equitable access to new therapies for OI as a RD. It also addresses the calls for European decision makers to maximise the value of what already exists within a robust methodological and quality-assured manner. It is based on prospective planning and collaboration with all key stakeholders (e.g., decision-makers, healthcare providers, treating physicians, registry managers, the company and, most importantly, the patient community that this approach is seeking to ultimately benefit).

In addition, the formation of the common core dataset will align various European collaborators on key data collection standards, which may contribute to future discussion around standards of care and an improved patient journey for people with OI both in Europe and globally, and learnings from which may also contribute to or be relevant for other RD areas.

### Electronic supplementary material

Below is the link to the electronic supplementary material.

Supplementary Material 1

## Data Availability

Not applicable.

## Abbreviations

CRF: Case Report Form
EC: European Commission
EMA: European Medicines Agency
ERN BOND: European Reference Network of Bone Diseases
EU: European Union
EUnetHTA: European Network for Health Technology Assessment
EU-RD Platform: European Platform on Rare Disease Registration
EuRR-Bone: European Registries for Rare Bone and Mineral Conditions
FDA: Food and Drug Administration
HTA: Health Technology Assessment
IOR: IRCCS Istituto Ortopedico Rizzoli
OI: Osteogenesis Imperfecta
OIFE: Osteogenesis Imperfecta Federation Europe
PAES: Post-authorisation efficacy studies
PASS: Post-authorisation safety studies
RDs: Rare Diseases
REQueST: Registry Evaluation of Quality Standards Tool
ROI: Registry of Osteogenesis Imperfecta
SATURN: Systematic Accumulation of Treatment practices and Utilization, Real world evidence and Natural history data in the rare disease Osteogenesis Imperfecta
TLFs: Tables, Listings and Figures
UK: United Kingdom
US: United States
