# Supplementary material for: Project SATURN– a real-world evidence data collaboration with existing European datasets in Osteogenesis Imperfecta to support future therapies
Source: Orphanet J Rare Dis. 2024 May 2;19:184. doi: 10.1186/s13023-024-03185-y (PMC11064334; doi:10.1186/s13023-024-03185-y)
Supplement: Supplementary file 1 — Supplementary Material 1 [file 13023_2024_3185_MOESM1_ESM.docx]

Reviewer reports:

Reviewer #1: Very well written paper with a potentially very useful clear concept that can benefit patients and other stakeholders significantly. Congratulations. For the general reader the role of the pharmaceutical company in this paper and in the project (authors and funding) might be better/more transparently described in the text of the paper (introduction/conclusion), especially explaining the benefits of this type of collaboration between a pharmaceutical company and the stakeholders like researchers, patients, patient organizations etc.

Response: The manuscript has been updated in the ‘Background’ section on page 4 with information with regards to funding of project SATURN and introduction of the various stakeholders.

The benefit of this collaboration is described at the end of section ‘The Need for a Common Core Dataset’ on page 6.

Reviewer #2: This is a well-presented summary of the SATURN project, but the manuscript does not include any data or results. Some clarification can be made to certain areas, see below.

Specific points in order of appearance:

1. Introduction: Can the authors please briefly describe what was the major shifts in the European Union's Regulation on orphan medicinal products that made the development of medicines for rare diseases accelerate?

Response: The manuscript has been updated in the ‘Background’ section at the end of page 2 with “sponsors of orphan medicinal products designated under EU Regulation 141/2000 should be entitled to the full benefit of any incentives granted by the Community or by the Member States to support the research and development of medicinal products for the diagnosis, prevention or treatment of rare diseases.”

2. Have a readthrough to streamline "rare diseases" vs rare conditions" vs "orphan diseases" etc throughout the manuscript.

Response: The manuscript has been streamlined to state ‘rare diseases’ throughout and has been consistent with terminology where possible but has been unable to do so where regulation is quoted. Only the use of ‘orphan medical products’ term was kept.

3. Can the authors define the difference between "Effectiveness" vs "Relative Effectiveness" data?

Response: Relative efficacy is the extent to which an intervention does more good than harm, under ideal circumstances, compared to one or more alternative interventions.

Effectiveness is the extent to which an intervention does more good than harm when provided under the usual circumstances of health care practice. ([DocsRoom - European Commission (europa.eu)](https://ec.europa.eu/docsroom/documents/7581?locale=en)).

The manuscript has been updated at the end of page 5 in the section ‘The Need for a Common Core Dataset’.

4. It would be better to explain the different Stakeholders earlier in the manuscript, and also to define the stakeholders in rare diseases, in OI and in the SATURN project specifically.

Response: The manuscript has been updated in the ‘Background’ section on page 4 with information with regards to funding of project SATURN and introduction of the various stakeholders.

5. The manuscript is somewhat repetitive, please read through and condense.

Response: The manuscript has been updated with several deletions in pages 6,7 and 8.

6. Are there any potential pitfall regarding data protection (i.e. the European GDPR act) for the project?

Response: Only aggregate level data will be shared, ensuring GDPR is followed. The manuscript has been updated on section ‘Pilot Study’ on page 11.

How is consent from the patients ensured for this specific project?

Response: Pseudonymized patient data, in accordance with national and European regulations, which allows sharing of pseudonymized aggregate level data without further consent. The manuscript has been updated on section ‘Pilot Study’ on page 11.

7. Who will be the owner of the SATURN dataset and who will get access to it and how?

Response: Each portion of SATURN core dataset will be owned by each registry that contributed to it. Mentioned in section ‘The Concept of Project SATURN’ on pg 8 ‘data-ownership still remaining at source in the hands of those who originally collected it and will continue to collect it.

Is there funding available for centres that would like to contribute with datasets to SATURN?

Response: Yes, collaborators will be compensated for their time and data collection. Manuscript has been updated on page11, section on ‘Further Collaboration to Include Datasets in Other European Countries’: ‘There is funding available for the collaborators’ time and data collection.’

8. Is it know what type of data variables that will be collected, and what will be age of the patients (adults vs paediatric)?

Response: Yes, however in this manuscript this level of information will not be shared. There are plans to publish a second manuscript that will contain variable level information and the results for pilot study. Age will be collected as part of patient characteristics, at aggregate level data. The hope is SATURN will have both adult and paediatric data included.

9. Will it be possible to add additional data from future follow-up visits from a specific patient to the dataset?

Response: The manuscript has been updated in section ‘Pilot Study’ on page 11 ‘As project SATURN progresses, there will be further data collections or transfers allowing the possibility to add additional data from future follow-up visits to the dataset. The initial transfer will be a natural history cut. It is hoped that as further data is collected and the shared data variables defined and complete, that longitudinal data will be collected from the collaborations to answer research questions in the future.’

10. What is the time plan for SATURN and what is the current status?

Response: The initial pilot for SATURN is planned for completion in 2024. Further collaborations are being assessed to begin work in 2024 as well. Manuscript has been updated on pages 11 and 12 section ‘Further Collaboration to Include Datasets in Other European Countries’.

11. Is there a tentative plan for "testing" the data in real life? Thinking of that one of the co-authors is from Mereo BioPharma.

Response: The dataset will be tested within the pilot with ROI, further tests will come when combining outputs from various datasets for the meta-analyses.

Reviewer #3: I think that this article describes an important attempt to deal with an issue that is of great significance to a variety of stakeholders. I am not aware of similar work that seeks to address the various barriers of getting new drugs to individuals with rare diseases across a broad range of jurisdictions. As such, this project is to be welcomed and it is important that a good description of the work is published. The description of the problem is very good but the paper does lack clarity and detail in some important aspects. Most of my comments relate to the latter part of the manuscript.

Specific comments:

Background

p2 line 46 - I think the 10% figure might more be more accurately said to refer to a "medical treatment option" rather than just "treatment option".

Response: The manuscript has been updated in section ‘Background’ on page2 with ‘medical treatment option’.

p3 line 14 - states that 50% of [orphan..products] were available. It would be helpful to clarify what is meant by "available".

Response: ‘Available’ means – centrally authorized orphan medicinal products however access to patients was further restricted by other national reimbursement policies. Manuscript has been updated in section ‘Background’ on pg3.

p3 line 19 - ".....ensuring quality of life, and accessing therapies at the root cause of the condition" - I think this needs rephrasing for clarity of meaning.

Response: Manuscript has been rephrase in section ‘ Background’ on page 3 to ‘…accessing therapies that are more targeted to the condition.’ .

The osteogenesis imperfecta community

p5 line 7 - "This was initially a three-year project..." To what does this refer? Is it EuRR-Bone? If so, could be more clear.

Response: Correct this referred to EuRR-Bone, manuscript has been updated in section ‘The Osteogenesis Imperfecta Community’ on page5.

The concept of Project SATURN

p7 line 19 - Objectives include avoidance of fragmentation and supporting equity. These seem to me to be aims rather than specific objectives. I also think that development of a common dataset alone cannot achieve these things. It would be helpful to have a clearer description of how the project might help achieve these particular objectives/aims.

Response: We did describe in section ‘Potential Challenges’ on page 7 : ‘The objective is to work with existing datasets, within a framework that corresponds to what the data-customers (e.g., regulators, HTAs, healthcare systems) will seek, avoiding fragmentation through multiple approaches (e.g., a series of individual national requests/approaches and unconnected with the regulators’ potential requirements) and supporting equity of access to potential new therapies for OI.’

Development of core dataset

p7 line 51 - Whilst core dataset said to correspond to main data fields "which all independent datasets" are collecting, I see this as a limitation as there may be elements which need to be in a core dataset which are not collected by some centres (there is a later reference to less formal datasets on p10).

Response: Agreed there could be elements that should be in the core dataset which are not collected by registry/site. This limitation is addressed in section ‘Potential Challenges’ on page9. This in turn will depend on each registry/site to see if they want to collect additional datasets and will be discussed individually.

Is it true that only data collected by "all" datasets would be included in a core dataset?

Response: No. Mentioned in section ‘Potential Challenges’ on page 9. However, there remains the possibility that a stakeholder may require data to be collected that is not in the existing dataset. This is particularly the case for adverse event data collection, which may not always be captured, especially when the focus of the data collection is clinical outcomes. Post Authorisation Safety Studies, by definition, will require the collection of adverse event data for future new therapies for OI.’

p8 line 18 - There is a description of gaps and building the dataset "based on these". On the face of it this contradicts the description of a core dataset that corresponds to fields collected by "all independent datasets". This may be a point that simply requires some clarification.

Response: We have a draft dataset, this will be refined as SATURN progresses and we understand what is collected by existing datasets. Additionally, if datasets are open to it, they can add further data collection variables to their datasets to meet the shared variables. Manuscript has been updated at the end of section ‘Development of core data set’ on page 8.

Potential challenges

p8 line 36 - "As a result, there is potential for missing data" - I wonder if it would be more accurate to state this more strongly e.g. that there is "likely to be" missing data. Also, the point above regarding conditions of clinical practice will probably result in erroneous as well as missing data and this is an important point to recognise.

Response: The manuscript has been updated in section ‘Potential Challenges’ on page 9 with ‘there is likely potential for missing data.’

p8 line 51 - "...data elements to be non-standardised...." - I think an example would be helpful.

Response: The manuscript has been updated in section ‘Potential Challenges’ on page 9 with ‘(i.e., quality of life questionnaires)’

Pilot study

p9 line 38 - Does the gap analysis compare ROI with general standards for databases or against a more specific standard?

Response: The gap analysis compares ROI variables against more specific standards. Manuscript has been updated on page10 ‘Pilot Study’ section.

p9 line 51 - It would be helpful to have more clarity about precise purpose of the gap analysis.

Response: The gap analysis’ purpose is to assess what data variables are collected by the registry/site. Manuscript has been updated on page10, ‘Pilot Study’ section.

p9 line 53 - It would be helpful to have an indication of what the critical variables are.

Response: The manuscript has been updated on ‘Pilot Study’ section page 10 ‘..against a list of critical variables (assessments, fracture collection and history, treatment for OI, treatment for pain, registry consent, safety, quality of life, patient characteristics, health resource utilization)..’

p10 line 9 - What data will be extracted from the IOR? Does this relate to the gap analysis or REQueST?

Response: Critical variables will be extracted from ROI. The manuscript has been updated in section ’Pilot Study’ page 10 listing the critical variables.

p10 line 11 - To whom will the TLFs be sent - is it the stakeholders? And who will send them?

Response: The manuscript has been updated on section ‘ Pilot Study’ page10 ‘ The results will be shared with stakeholders through Mereo BioPharma.’

Are there any results of the gap analysis and REQueST available? It appears from text that this has been done and so it would be helpful to have some description of findings, even if provisional and general.

Response: Yes, however they will be shared in a separate manuscript.

Further collaboration

p10 line 18 - It would be helpful to be explicit about what process will be repeated - is the gap analysis, REQueST, and data extraction?

Response: The manuscript has been updated on section ‘Further Collaboration to Include Datasets in Other European Countries’ on page11 ‘ The same process (variable gap analysis and REQueST assessment) will then be repeated …’.

How will it be determined whether centres are "sufficiently robust"?

Response: The results of the variable gap analysis and REQueST assessment will determine if data is robust for Project SATURN. Page 11, Section “Further Collaboration to Include Datasets in Other European Countries” has been updated to explain this point.

p10 line 26 - Which elements of the approach might be adapted and how would this impact on validity of overall project? Description of a retrospective chart review for "less formal" datasets seems to suggest that approach is about data extraction rather than a review of content/gaps/quality of dataset. It would be helpful for scope of all aspects of project to be described more clearly.

Response: The adaptation mentioned on page 10 will not impact validity of the project or the robustness of data. The gap analysis and REQueST assessment is still to be done. Less formal/smaller datasets may not have the team or system in place to create aggregate TLFs. Therefore, adaption will be needed to obtain such data, i.e., chart abstraction. The manuscript has been updated on section ‘Further Collaboration to Include Datasets in Other European Countries’ page.11 ‘The approach to assisting each registry or site will be adapted based on the existing dataset …’

p10 line 33 - "...regardless of methodology, all data will be assessed in the same way..and all data will remain at source...". It would be helpful to clarify who is undertaking assessment. It would also be helpful to clarify what is meant how assessment is distinct from methodology.

Response: The manuscript has been updated in section ‘Further Collaboration to Include Datasets in Other European Countries’ page11 ‘ However, regardless of the data abstraction methodology (chart review, aggregate TLF transfer), all data will be assessed in the same way (using the core dataset for variable gap analysis and REQueST assessment) by Mereo BioPharma and all data collected will remain at source within the community.’

p10 line 38 - Paragraph starting "This approach will be progressed gradually....". I think this important paragraph lacks clarity.

Response: The manuscript has been updated on section ‘Further Collaboration to Include Datasets in Other European Countries ‘page 11 to ‘… this approach will be applied gradually in more countries to build a European dataset ….’

p10 line 55 - How will local data collection be supported?

How will the project be funded?

Response: The collaboration is being undertaken with existing datasets therefore funding is likely already in place locally. Mereo BioPharma will support financially for the collaboration, compensate for data collection. Manuscript updated in page11 section named ‘Further Collaboration to Include Datasets in Other European Countries’.
